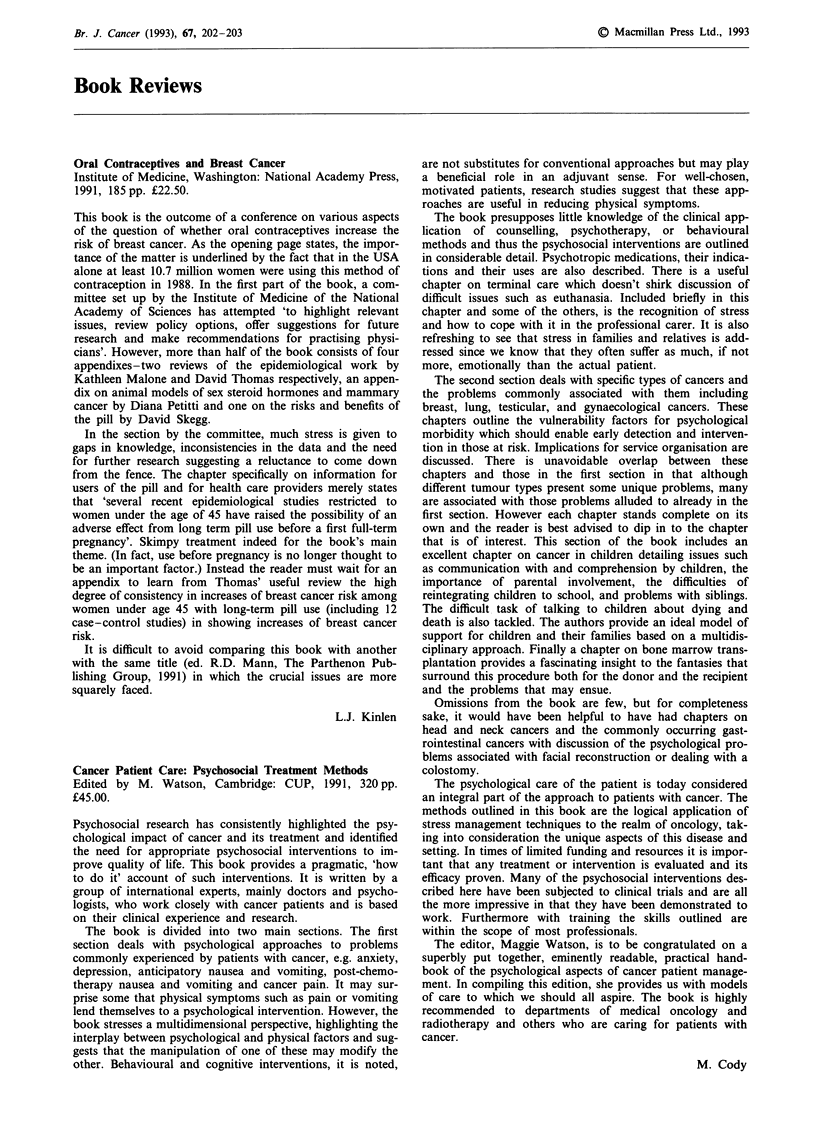# Oral Contraceptives and Breast Cancer

**Published:** 1993-01

**Authors:** L.J. Kinlen


 © Macmillan Press Ltd., 1993

### Book Reviews

**Oral Contraceptives and Breast Cancer**
Institute of Medicine, Washington: National Academy Press, 1991, 185 pp. £22.50.

This book is the outcome of a conference on various aspects of the question of whether oral contraceptives increase the risk of breast cancer. As the opening page states, the importance of the matter is underlined by the fact that in the USA alone at least 10.7 million women were using this method of contraception in 1988. In the first part of the book, a committee set up by the Institute of Medicine of the National Academy of Sciences has attempted 'to highlight relevant issues, review policy options, offer suggestions for future research and make recommendations for practising physicians'. However, more than half of the book consists of four appendixes–two reviews of the epidemiological work by Kathleen Malone and David Thomas respectively, an appendix on animal models of sex steroid hormones and mammary cancer by Diana Petitti and one on the risks and benefits of the pill by David Skegg.

In the section by the committee, much stress is given to gaps in knowledge, inconsistencies in the data and the need for further research suggesting a reluctance to come down from the fence. The chapter specifically on information for users of the pill and for health care providers merely states that 'several recent epidemiological studies restricted to women under the age of 45 have raised the possibility of an adverse effect from long term pill use before a first full-term pregnancy'. Skimpy treatment indeed for the book's main theme. (In fact, use before pregnancy is no longer thought to be an important factor.) Instead the reader must wait for an appendix to learn from Thomas' useful review the high degree of consistency in increases of breast cancer risk among women under age 45 with long-term pill use (including 12 case–control studies) in showing increases of breast cancer risk.

It is difficult to avoid comparing this book with another with the same title (ed. R.D. Mann, The Parthenon Publishing Group, 1991) in which the crucial issues are more squarely faced.

L.J. Kinlen